# Happy Holidays and Happy New Year

**DOI:** 10.31486/toj.24.5051

**Published:** 2024

**Authors:** Ronald G. Amedee

**Affiliations:** Director of Clinical School and Professor, The University of Queensland Medical School, Ochsner Clinical School, New Orleans, LA; Editor-in-Chief, *Ochsner Journal*

*You can tell a lot about a person by the way they handle three things: a rainy day, lost luggage, and tangled Christmas tree lights.*

*–Maya Angelou*

The Winter 2024 issue of the *Ochsner Journal* contains three original research articles and seven case reports and clinical observations. In addition, a must-read is the quarterly submission of the Health, Medicine, and Society column authored by Ochsner academic physician Dr Tamika Webb-Detiege entitled “The Disappearance of Black Men From Medicine: A Consequence of Racism and the Flexner Report” which provides a thought-provoking personal lens to view this issue through. This is followed by two editorials, the first of which, penned by Ochsner academic champion Dr Yvens Laborde, is “The Xavier Ochsner College of Medicine – ‘The Time Is Always Right To Do What Is Right’ ” which serves as a complementary endorsement of many of the comments expressed by Dr Webb-Detiege in the previous article. Finally, Ochsner research academician, Dr Richard Re, who retired from Ochsner Health this past summer, offers an interesting historic review in “A Look Back at the Second Generation of Ochsner Research.”

“MedVantage: A Primary Care Model for Populations With High Social and Medical Needs” authored by local medical students Shawley, Jung, Lim, and Kavanagh, as well as Ochsner Clinical School faculty Denton and Carstarphen and other coauthors, is the first original research article in this issue. The second original research article “I’m Getting a Migraine: A Comparative Evaluation of Patient and Clinician Interpretations of Migraine Symptoms” was submitted by Fischer, Tolisano, Navarro, Abuzeid, Humphreys, Akbar, Shah, Schneider, Riley, and McCoul (from Ochsner Otolaryngology). Next in this section, we find an article by Hajirawala, Hardeman, Hein, and Carlson (from Ochsner Pediatrics) on “A Model for Consolidating High-Risk Allergy Procedures in Clinic.”

Case reports and clinical observations include “Among the Masses: Multiple Left Atrial Undifferentiated Pleomorphic Sarcomas” by Yager, Fan, Sanford, Pourfarrokh, and Nguyen, followed by the next report entitled “Waldenström Macroglobulinemia–Induced Cardiac Amyloid Light Chain Amyloidosis” authored by Fan, Chukwu, Sanford, Jebakumar, Quitoriano, and Nguyen. Next, we have “Heterotaxy Syndrome Diagnosed in an Adult” submitted by Guney, Aydinli, Aksit, Kadirli, and Salmanoglu, followed by a report on “Trident Sign: The Key Magnetic Resonance Imaging Finding Distinguishing Spinal Cord Sarcoidosis From Multiple Sclerosis and Seropositive Neuromyelitis Optica Spectrum Disorder” contributed by the Ochsner team of Beitollahi, Berry, Gulotta, Morales, and Milburn. Barasker, Jain, Gautam, and Saxena discuss “Ultrasonography- and Fluoroscopy-Guided Technique for Cooled Radiofrequency Ablation of the Genicular Nerves for Knee Joint Pain.” “Rare Prekallikrein Deficiency Identified During Workup of Isolated Prolonged Activated Partial Thromboplastin Time” authored by Woo, Tseng, Kang, Zhuang, Jackson, Danilova, and Borogovac is followed by our final report from Taha, Carroll, and Gehlert discussing a “Novel Treatment for a Completely Extruded Talus.”

As we close out this calendar year, I would like to wish you all a joyous holiday season and a healthy and prosperous New Year 2025. The *Journal* would not be possible without the expertise and time commitment of our authors and reviewers, and without Kathleen McFadden our Managing Editor, Publishing Services, Ochsner Health, who is the guiding force behind the accomplishments achieved by this publication. Thank you all, Happy Holidays, and see you once again in the New Year!

